# Cluster Randomized Controlled Trial Evaluation of a Gender Equity and Family Planning Intervention for Married Men and Couples in Rural India

**DOI:** 10.1371/journal.pone.0153190

**Published:** 2016-05-11

**Authors:** Anita Raj, Mohan Ghule, Julie Ritter, Madhusudana Battala, Velhal Gajanan, Saritha Nair, Anindita Dasgupta, Jay G. Silverman, Donta Balaiah, Niranjan Saggurti

**Affiliations:** 1 Center on Gender Equity and Health, Division of Global Public Health, University of California San Diego School of Medicine, San Diego, CA, United States of America; 2 National Institute for Research in Reproductive Health (NIRRH), Mumbai, India; 3 Population Council, New Delhi, India; 4 T N Medical College & B Y L Nair Ch Hospital, Mumbai, India; University of Toronto Dalla Lana School of Public Health, CANADA

## Abstract

**Background:**

Despite ongoing recommendations to increase male engagement and gender-equity (GE) counseling in family planning (FP) services, few such programs have been implemented and rigorously evaluated. This study evaluates the impact of CHARM, a three-session GE+FP counseling intervention delivered by male health care providers to married men, alone (sessions 1&2) and with their wives (session 3) in India.

**Methods and Findings:**

A two-armed cluster randomized controlled trial was conducted with young married couples (N = 1081 couples) recruited from 50 geographic clusters (25 clusters randomized to CHARM and a control condition, respectively) in rural Maharashtra, India. Couples were surveyed on demographics, contraceptive behaviors, and intimate partner violence (IPV) attitudes and behaviors at baseline and 9 &18-month follow-ups, with pregnancy testing at baseline and 18-month follow-up. Outcome effects on contraceptive use and incident pregnancy, and secondarily, on contraceptive communication and men’s IPV attitudes and behaviors, were assessed using logistic generalized linear mixed models. Most men recruited from CHARM communities (91.3%) received at least one CHARM intervention session; 52.5% received the couple’s session with their wife. Findings document that women from the CHARM condition, relative to controls, were more likely to report contraceptive communication at 9-month follow-up (AOR = 1.77, p = 0.04) and modern contraceptive use at 9 and 18-month follow-ups (AORs = 1.57–1.58, p = 0.05), and they were less likely to report sexual IPV at 18-month follow-up (AOR = 0.48, p = 0.01). Men in the CHARM condition were less likely than those in the control clusters to report attitudes accepting of sexual IPV at 9-month (AOR = 0.64, p = 0.03) and 18-month (AOR = 0.51, p = 0.004) follow-up, and attitudes accepting of physical IPV at 18-month follow-up (AOR = 0.64, p = 0.02). No significant effect on pregnancy was seen.

**Conclusions:**

Findings demonstrate that men can be engaged in FP programming in rural India, and that such an approach inclusive of GE counseling can improve contraceptive practices and reduce sexual IPV in married couples.

**Trial Registration:**

ClinicalTrials.gov NCT01593943

## Introduction

Globally, an estimated 153 million women contend with unmet need for family planning (FP); more than one in five of these women reside in India [1]. Inadequate FP progress in India has been attributed to over-reliance on female sterilization as the preferred, and often only, means of modern contraception used, and low female control over contraception, particularly among young and rural married women [2]. Awareness of modern spacing contraceptives, i.e., contraceptives designed to impede fertility on a reversible basis, is high in India, even among young, less educated and rural women, and availability of these contraceptives at low and no cost through India’s public health care system is also well understood [2]. Nonetheless, of the 49% of married and sexually active unmarried women of childbearing age who use modern contraceptives in India, fewer than one in four (22.9%, or 11.1% of all of these women) use spacing contraceptives, such as oral contraceptive pills (6.4%, or 3.1% of all of these women), condoms (10.7%, or 5.2% of all of these women), and intrauterine contraceptive devices (3.5%, or 1.7% of all of these women) [2]. Research from India indicates that family planning programs often attempt to reach young wives only after they reach their family size goal, despite indications that demand for contraceptive use to delay first pregnancy is high [3]. Low support for and use of spacing contraceptives to delay first pregnancy and improve birth spacing contribute to ongoing high rates of infant mortality [4] and one in five unintended pregnancies in India [2].

Increasing use of modern spacing contraceptives in the country requires improved demand for such contraception from both men and women, given that men often control contraceptive decision-making [5–13]. Male reproductive control of female partners, particularly in the context of intimate partner violence (IPV), can impede contraceptive use and increase risk for contraceptive failure, in India and globally [14–16]. Given men’s role in controlling contraception and, relatedly, the need to improve gender equity in this context (as demonstrated by disproportionate burden of IPV in the country [2, 17]), engagement of men in FP interventions requires greater prioritization. Unfortunately, no model of FP promotion with male engagement has been rigorously evaluated for India; the single rigorously evaluated FP intervention that has engaged men was conducted in Malawi and did not assess for effects on men’s IPV perpetration [12]. The current study evaluates CHARM [Counseling Husbands to Achieve Reproductive health and Marital equity], a three-session gender equity and family planning (GE+FP) counseling intervention delivered by male health care providers to married men, both alone and with their wives, to improve contraceptive use and reduce incident pregnancy and, secondarily, improve contraceptive communication and reduce IPV perpetration and acceptability in rural India.

## Methods

A two-armed cluster randomized controlled trial was conducted to evaluate the impact of the CHARM intervention on marital contraceptive use and incident pregnancy, and secondarily on contraceptive communication and men’s IPV attitudes and perpetration. Married couples (N = 1081) were recruited from rural areas of Thane district, Maharashtra, India from March to December 2012, and followed over a period of 18 months for this evaluation trial. Rural Thane district was selected to be the study setting due to its prevalence of high and early fertility, low contraceptive use, high unmet need for family planning and limited access to government health services; the area is also characterized by high representation of tribal communities. This selection was made by our partnering medical college and government agencies based on non-public data.

### Randomization and Masking

Participating couples were recruited from 62 geographic clusters of approximately equal size mapped for the purpose of randomization. Clusters were created based on geographic boundaries, population density (approximately 300 households per cluster), and proximity to public and private health services. Fifty of the 62 clusters were selected based on ease of reach, then randomized to intervention or control conditions using computer-generated random numbers. Clusters were geographically distinct areas with natural borders and sufficient distance from other study clusters to reduce risk for contamination. Clusters were randomized into intervention and control conditions on 20^th^ Feb 2012, in the month prior to initiation of enrollment. Households within each cluster were screened sequentially for eligibility; recruitment of eligible households was capped at n = 25 per cluster. Neither participants nor research staff were masked to the treatment condition.

### Participants

Participants of this two-armed design (n = 467 intervention condition couples, n = 614 control condition couples) were surveyed at baseline and 9 and 18-month follow-up (with a window of no more than one month around the due date); women were tested for pregnancy at baseline and at 18-month follow-up. Eligible couples included husbands aged 18–30 years and their wives. Although the legal age of marriage is 21 for males and 18 for females in India, marriage of minors is not uncommon [2]. Participants were required to be fluent in Marathi (native language of Maharashtra) and residing together for the past three months with no intent to relocate in the next 2 years. Couples reporting infertility, surgical sterilization, or exhibiting serious cognitive or health impairment were excluded. Both members of the couple had to provide consent and indicate eligibility and willingness to participate in this study. Of the 1881 participants approached to assess eligibility, 1162 were identified as eligible, and 1081 of these agreed to participate in the study (81 declined, 93% participation rate). The very high participation rate is attributable to the fact that participants would only allow us to assess for eligibility if they were open to participation.

### Sample Size Determination

Sample size and power considerations were constructed based on our primary outcomes of interest: any spacing contraceptive use and communication, and assuming a baseline sample size of 1000 couples, equally distributed across the 50 clusters (25 intervention clusters and 25 control clusters). We also assumed 80% retention by 18-month follow-up and thus, based our calculations on 800 men. All calculations were based on 2-sided logistic regressions with a significance level of 0.05. While we utilized longitudinal regression methods in our analyses, power calculations were more conservatively based on single time-point methods. Computations were also adjusted for the design effect in order to account for the correlation of subjects within the same village. Assuming 20 men enrolled in each village, and a within village correlation (Kappa) of 0.10, the design effect [1 + within village correlation*(# of men per village-1)] was estimated to be equal to 2.9. Details on power calculations for primary outcomes can be found in Yore et al., 2016 [18]. However, we also provide an example calculation specific to one of our primary outcomes, use of modern spacing contraceptives.

Power calculations for this binary outcome were based on logistic regression of the outcome on a binary independent variable (i.e. Group 1 = Intervention Group, Group 2 = Control Group) with 800 subjects equally distributed between the two groups. If the proportion of women reporting use of contraceptives in Group 2 is 8%, 10%, or 12%, then we would have over 80% power to detect a difference between Group 1 and Group 2, when the corresponding proportion in Group 1 is 20% (OR_Group1/Group2_ = 2.8), 22% (OR_Group1/Group2_ = 2.59), and 25%(OR_Group1/Group2_ = 2.45), respectively. Based on the NFHS-3 data [2], we expected that the proportion of couples using any marital spacing contraception in the control group (Group 2) would be 10% at 18 months. Based on the above assumptions, the proposed study had 80% power to detect an absolute difference as small as 12% between the two groups.

### Study Retention

**Fig 1:**
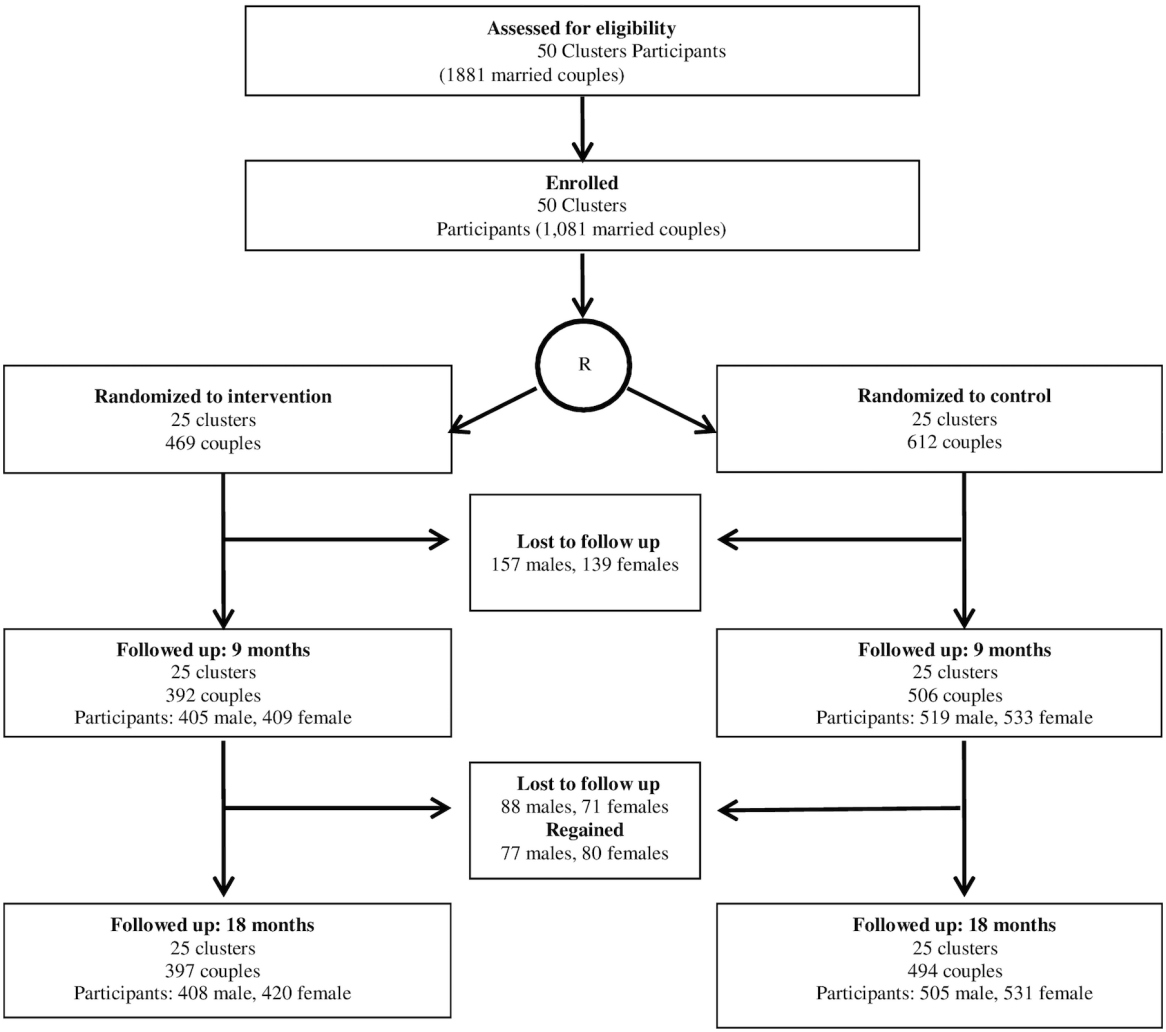
CHARM Consort Figure.

Of the 1081 couples participating in baseline assessment, 83.1% (n = 898) and 82.4% (891 couples) completed 9 and 18 month follow-ups, respectively. [See Fig 1] Reasons for loss to follow-up were predominantly inability to find participants due to relocation, or refusal due to time constraints. No one withdrew from the study. All available data were included in analyses.

### Procedure

Trained male-female research teams approached identified households for recruitment. Age-eligible couples indicating interest and willingness to participate provided written informed consent and were screened privately for eligibility. Once eligibility was ascertained, couples privately and separately participated in the baseline survey, and women provided urine for pregnancy testing. Surveys were administered in an interview format with research staff asking participants all questions and noting their responses. With recognition of the high rates of spousal violence in India [2], World Health Organization guidelines for research on domestic violence were followed to help ensure that women participating in our study, which did include domestic violence assessments, were not at increased risk for violence due to their participation [19]. Hence, in addition to separately and privately surveying husbands and wives, we only surveyed women on experiences of spousal violence. We also did not inform husbands of the pregnancy test or test results obtained through this study. All participants, subsequent to baseline assessment, were provided with basic information regarding family planning and local public health system family planning services. All female participants were also provided with information on how to contact police and the nearest domestic violence services, which unfortunately were not local (two hours away by car); transport was to be offered to any participants indicating interest in linkage to those services, but no interest was indicated.

Following the baseline assessment protocol, husbands from intervention clusters were linked to male village health care providers trained to implement the CHARM intervention. Follow-up surveys were conducted again at 9 and 18-month follow-ups; urine tests for pregnancy were conducted only at 18-month follow-up. Although screener and baseline survey data were collected on paper, follow-up survey data were collected on mobile tablets presenting English and Marathi simultaneously, to ensure clarity of the questions for our bilingual research staff, using the MSHARE (Mobile Survey for Health Assessment, Research and Evaluation) System. The MSHARE system reduced human error in data collection and entry and allowed for real time data uploads and simultaneous data sharing on the internet, as well as more rapid management of data for analyses. Surveys and pregnancy testing required approximately one hour from participants. No monetary incentive was provided for study or program participation. For further details on procedures, see the study protocol paper [18].

All procedures were reviewed and approved by the Institutional Review Boards of University of California at San Diego, Population Council and India’s National Institute for Research in Reproductive Health. This study was registered with clinicaltrials.gov on May 2, 2012 (Clinical Trials number: NCT01593943), though study enrollment started on March 26, 2012. The delay in registration is due to our ignorance of this requirement for journal submissions from the study; we registered the trial soon after learning of the requirement. Only 172 couples, 16% of our final sample of N = 1081 couples, had been recruited prior to trial registration, and no analysis of data by treatment group was conducted at that point. Also, no changes to the study design or approach occurred during the period of study initiation and registration, such that the delayed trial registration was in no way related to or affected by the study. We confirm that all related trials to this intervention were registered; there are no ongoing trials related to this study.

### CHARM Intervention

The intervention involved three gender, culture and contextually-tailored family planning and gender equity (FP+GE) counseling sessions delivered by trained male village health care providers to married men (sessions 1 and 2) and couples (session 3) in a clinical setting, or if required, near or in the participant’s home. (See Table 1 for details on curriculum content by session.) A desk-sized CHARM flipchart was used by village health providers to provide men and couples with pictorial information on family planning options, barriers to family planning use including gender equity-related issues (e.g., son preference), the importance of healthy and shared family planning decision-making, and how to engage in respectful marital communication and interactions (inclusive of no spousal violence in the men’s sessions). Sessions were delivered over a three month period. [See Table 1 for details.] At each contact, village health providers also offered free condoms and oral contraceptive pills; the latter required women to visit the provider. Formative research for the study indicated that retention of men across multiple sessions and couple participation in sessions might prove difficult. Hence, session 1 was designed to include all required content, in case participants were then lost to program, and sessions 2 and 3 could be optional. Sessions 2 and 3 were not presented to the participants as optional in practice.

**Table 1:** Intervention modules and delivery schedule.

Session	Content focus	Strategies
Individual Session 1 (Male)	Assess client’s FP* knowledge and goals; provide overview of FP options and their availability.	Assessment
	Assess man’s fertility goals- desire for more children, planned timing for (more) children, expectations of children early in marriage or sons; consider role and expectations of parents	Dialogue
20–40 min	Provide info on maternal and child health benefits of contraception and birth spacing, as well as delayed first childbirth, particularly for adolescent wives	Education
	Assess sex risk of man: extramarital sex; provide basic HIV/STI prevention information	FP Goal Setting & Action Plan
	Briefly assess if man has discussed FP with wife; assess & encourage joint FP decision-making	Provision of Condoms and/or Pill
	Highlight importance of male involvement in FP, safe motherhood and happy family life.	
	Review again client’s FP goals; offer condoms, encourage consideration of pill	
Individual Session 2 (Male)	Assess client’s FP goals; review FP options to support these goals	Assessment
	Review previously identified barriers to FP uptake- desire for more children or for sons, expectations of parents, negative attitudes toward contraception; Process barriers with client	Dialogue
20 min	Assess if man has discussed FP with wife; practice how to communicate about FP with wife	Education
	Assess marital violence and sexual communication; reinforce non-use of violence and respectful communication; encourage joint FP decision making with wife	FP Goal Setting & Action Plan
	Highlight importance of male involvement in FP, safe motherhood and happy family life.	Provision of Condoms and/or Pill
	Review again client’s FP goals; offer condoms, encourage consideration of pill	
Couple Session 3	Assess couple’s FP goals; review FP options to support these goals	Assessment
	Discuss barriers to FP uptake- desire for more children or for son (son preference), expectations of parents, negative attitudes toward contraception; Process barriers with couple	Dialogue
	Assess joint decision-making on FP; support joint communication on FP; respect for wives	Education
20 min	Highlight importance of male involvement in FP, safe motherhood and happy family life.	FP Goal Setting & Action Plan
	Review again couple’s FP goals; offer condoms and pill	Provision of Condoms and/or Pill

*FP = Family Planning

Note: To be delivered in a three month timeframe, ideally.

#### CHARM Theoretical Framework

The CHARM intervention was developed based on a theoretical framework inclusive of Social Cognitive Theory (SCT) [20] and Theory of Gender and Power (TGP) [21]. SCT has been used in effective family planning interventions [22] and posits that behavior change, in this case contraceptive use, is more likely if an individual perceives positive outcomes for engaging in the behavior (e.g., beliefs that spacing births through contraception will produce healthier children), feels capable of engaging in and controlling the behavior (i.e., self-efficacy to use contraception), and has an environment supportive of the behavior (e.g., access to FP services). Hence, SCT would support use of FP education and skills building with improved access to contraceptives. TGP is a social-structural theory that posits that gender-based power dynamics inherent to many heterosexual dyadic relationships due to societally reinforced social norms can facilitate male control over sexual and reproductive decision-making, including contraceptive use, and some men may even use violence to control their female partners. Hence, counseling that can affect gender-equity and FP normative beliefs among men, particularly if the counseling was delivered by a respected male, could be useful in improving contraceptive use in the context of safer and healthier relationships.

#### CHARM Development

The CHARM model and curriculum was developed by the research team, which included social scientists and public health experts from India and the U.S. with experience in intervention design and expertise in the areas of FP, gender-based violence prevention, gender equity counseling, and male-focused health interventions. CHARM included Government of India FP information, education and counseling (IEC) materials used in the public health system to provide basic FP knowledge and positive outcome expectancies as well as contraceptives [23]. Additional elements focused on gender equity and social norms were then created for the CHARM curriculum based on the above described theoretical framework and findings from formative research. Formative research involved qualitative data collection from rural couples, mothers-in-law, and providers serving rural couples. Findings demonstrated the need for FP education to dispel ongoing myths related to health consequences of spacing contraceptives and GE social norm change approaches related to expectations of pregnancy early in marriage, son preference, lack of male responsibility in FP, and greater male or in-law relative to female control of FP decision-making [24]. Although not published, this research also documented that males and females preferred not to engage mothers-in-law in the intervention process. We created the intervention to allow for in-law inclusion in the couple session if the participants wished this inclusion, but no couples included in-laws in their sessions.

The three session CHARM model was developed to provide FP+GE counseling within a short timeframe, three sessions within three months, due to limited access to working men. Two sessions were included for men to allow rapport-building for discussion of more sensitive topics such as spousal violence in session 2. The couple session was designed to reinforce messaging to men and support joint family planning decision-making, and to facilitate reach to women for contraceptive pills. Once the model and curriculum were developed, review and feedback was obtained from rural health practitioners and FP experts for finalization. CHARM was then pilot-tested with ten couples, and pilot findings suggesting difficulty with retention resulted in our altering the sessions to provide all core FP+GE information (i.e., contraceptive options and safety, importance of joint and respectful FP decision-making among couples) in session 1 to allow sessions 2&3 to be optional, though the program was presented to participants as three sessions.

#### CHARM Providers

Clinics were not established through this study but rather the project engaged providers already practicing in the CHARM assigned areas and trained them to implement the CHARM program. CHARM providers were allopathic (n = 9) and non-allopathic (n = 13) village health care providers trained over three days on FP counseling, GE and IPV issues, and CHARM implementation. The FP counseling training was the standard public health FP training for the state, provided by our partnering medical college. Two half-day booster sessions were delivered within 3 months of the initial training, primarily focused on GE elements, based on observations suggesting the need for more training in this area. All trainings were conducted by a combination of academic physicians and researchers in India with expertise on FP, GE, and marital violence. Village health providers were selected to include private as well as public practitioners, based on private village health providers’ greater availability in the villages. This approach of using village health providers was based on the premise of extending public health services (e.g., family planning supervision/training and contraception options) via the private village health providers, in order to support public-private partnerships for family planning delivery. As most private village health providers are male, the CHARM intervention was designed to involve men reaching men to improve family planning, and simultaneously, offer more local family planning access. Public practitioners in CHARM would have received formal FP training prior to participation, but private providers may not; thus, comprehensive public health FP training was provided to all CHARM providers. Providers maintained case files and session checklists to monitor caseloads; clinical supervision and file reviews were provided in-person by senior clinicians every month. The private village health providers were paid 50 Indian rupees (approximately US$1.00) per CHARM session provided, as compensation for their time. The government village health providers were not given additional compensation but were guided to view their participation as part of their public health job, since the program was administered through the public health system in partnership with a local medical college.

### Control Condition

Women in the control condition were referred to government health system FP services, which provide no-cost contraception and home-based visits for family planning counseling and services delivered by front line public health workers (e.g., accredited social health activists- i.e., community outreach workers; the auxiliary nurse midwife). The control condition did not include use of village health providers, or any providers, to engage men in FP services, as that is not the standard of care in India.

### Measures

Sociodemographics (e.g., age, education- any and number of years) were assessed for both husbands and wives via single item measures. For those items shared by the couple (e.g., number of living sons and daughters), women’s data were used unless otherwise indicated. Women’s reports of contraceptive behaviors have been viewed as a reliable indicator for the couple within the context of India [25]. Pregnancy intent was assessed by asking women if they wished to have another child: now/soon, never, or within a specified number of months. Those reporting wanting another child now/soon or within 12 months were defined as having pregnancy intent.

#### Outcome Variables

Modern contraceptive use was assessed via a single item asking which form of contraception, if any, they had used in the past three months: pills, IUD, injectables, male or female condoms, or male or female medical sterilization (only in the follow-up surveys). Women reporting use of any listed method in the past three months were defined as using modern contraception.

Marital contraceptive communication was assessed via a single item to women inquiring whether they had discussed contraceptive use with their husband in the past three months.

Pregnancy was measured via self-report at all three time points and via urine testing for human chorionic gonadotropin (HCG) at baseline and 18 months. Self-reported pregnancy was included as an outcome at 9&18 month follow-ups, and a combined pregnancy variable including self-report and HCG test results data was used as an outcome at 18 month follow-up. Intendedness of identified pregnancies was not directly assessed at time of pregnancy. However, as noted above, at each assessment, women were asked if they wished to have another child: now/soon, never, or within a specified number of months. Hence, for the purpose of exploratory analyses, we defined an unintended pregnancy at 9-month and 18-month follow-up based on the female participant being pregnant at that time point but having reported no intent to have another child soon/now or in the next 12 months at their prior assessment.

IPV behavioral assessments were those included in India’s Demographic and Health Survey [2]. Physical IPV was assessed via six items regarding experiences of abuse in the past six months; the outcome was dichotomized based on endorsement of any item. Cronbach’s alpha for this six-item measure was 0.97. Sexual IPV was assessed via two items of past six month experiences, dichotomized based on a yes response to either item. Cronbach’s alpha for this two item measure was 0.97. [See Table 1 notes for specific IPV measure items.] As noted above, these items were taken only from the women.

IPV attitudinal assessments were also from India’s Demographic and Health Survey [2]. Men’s Attitudes regarding Acceptability of Physical IPV were assessed via a seven item measure in which men were asked if “a husband is justified in hitting or beating his wife” in specified situations; responding yes on any of these items was defined as having attitudes accepting of physical IPV. [See Table 1 notes for items.] Cronbach’s alpha for this seven item measure was 0.77. Men’s Attitudes regarding Acceptability of Sexual IPV were assessed via a single yes/no item asking men “if a woman refuses to have sex with her husband when he wants her to, does he have the right to get angry and reprimand her?”

#### Independent Variables

The primary independent variable was treatment group, CHARM or control. Dose analyses were also conducted in which intervention participants were classified as receiving: no session attendance, only male session attendance (one or both), and male and couple session attendance. No participants attended the couple session without previously attending at least one male-only session. Of 469 CHARM-assigned participants, 8.7% (n = 41) attended no sessions; 38.8% (n = 182) attended only male sessions, and 52.5% (n = 246) male and couple sessions. Control participants were included in the no session category.

#### Participant Satisfaction with CHARM Intervention

Men who had received at least one CHARM session were also eligible to participate in a brief survey on FP content and perceived utility of sessions as part of the 9 month follow-up assessment, to assess their perceptions of curriculum and response to program. Those eligible and unable to be surveyed at 9 month follow-up were asked to participate in the survey as part of their 18 month follow-up. Of the n = 428 men eligible to participate in this survey, based on their participation in at least one CHARM session, 347 (81%) provided responses to this survey.

### Outcome Analyses

Bivariate analyses in the form of t-tests and chi-squares were conducted to assess differences on demographics and outcomes at baseline: 1) by treatment group, 2) for those lost to study follow-up, and 3) for CHARM participants who participated in no sessions. Any characteristics identified as significantly different between groups were considered as potential covariates in respective adjusted models.

The two primary outcomes of interest, marital contraceptive use and incident pregnancy, as well as the secondary outcomes of marital contraceptive communication and IPV perpetration behavior and attitudes, were assessed via logistic regression generalized linear mixed models (GLMMs), using cluster as a random effect and with time, treatment group (CHARM vs control), and the time by treatment interaction as fixed effects. Covariates were included in the final adjusted models if they either were 1) selected a priori for inclusion due to being a known confounder or covariate, or 2) if significant baseline differences existed between assigned treatment groups and the variable maintained significance in the adjusted regression model. Models on contraceptive use and communication, pregnancy, and IPV outcomes adjusted for women’s age and education (any or none), caste or tribe, number of living sons, and number of living daughters, as well as pregnancy intent (contraceptive use and communication models only). Adjusted models for IPV attitude outcomes controlled for men’s age and education, and caste or tribe. A p<0.15 assessed significant time by treatment interactions [26]. Simple main effects were reported in order to describe the size of differences over time and between groups. All other analyses were evaluated for significance at p<0.05.

All analyses included both intent to treat and dose analyses approaches and were conducted using SAS (SAS Institute, Version 9.4, Cary, NC, USA). The GLIMMIX procedure in SAS was used to build the above described logistic generalized linear mixed models. This procedure allows for both G-side and R-side covariance structures. In these models, "cluster" (used at the level of randomization) was considered a random effect with a G-side covariance structure, while the variance resulting from repeated measures on each study participant over time was considered as a random effect with an R-side covariance structure. In both cases, a standard variance component (i.e., simple diagonal) was used for the underlying structure.

Additional exploratory chi-square analyses were conducted to assess differences between treatment groups at 9 and 18 month follow-ups on the contraceptive communication outcome, for the total sample and for the subsamples who did and did not report contraceptive communication at baseline, to assess whether observed effects on this outcome were attributable to new or continuing conversations. Exploratory chi-square analyses were also conducted to assess differences between treatment groups and by dose for the outcomes of pregnancy and unintended pregnancy across both follow-up time points, to allow for more opportunity for observation of pregnancy. These analyses were conducted to help clarify outcome analyses related to incident pregnancy and contraceptive communication, with findings included in supplemental tables.

## Results

Participants (N = 1081 couples) were majority (68.0%) tribal, and 17.5% of women and 8.5% of men had no formal education. Most (76.9%) had children; 42.3% reported marital contraceptive communication. (See Table 2) Excluding the 22.1% pregnant at baseline, 28.4% were using modern contraceptives. Significant differences were observed between intervention and control cluster participants at baseline for women’s education (intervention: 87.6%, control: 78.6%), and income (intervention: 6054 rupees, control: 4978 rupees) (p<0.05). These factors were included in the adjusted outcome analyses. No significant differences were seen between those who were retained and those who were lost to follow-up in the study, nor between CHARM participants that did and did not attend any counseling sessions.

**Table 2:** Characteristics of CHARM participants for the total sample and by treatment condition (N = 1081 couples).

	Total Sample	Intervention	Control	
	(N = 1,081)	(n = 469)	(n = 612)	
	% (n)	% (n)	% (n)	p-value^1^
Wife’s age [mean (SD)	22.5 (2.5)	22.7 (2.5)	22.4 (2.4)	0.025
Husband’s age [mean (SD)]	26.2 (1081)	26.4 (2·6)	26.0 (2.7)	0.012
Wife ever attended school	82.5 (892)	87.6 (411)	78.6 (481)	0.0001
Husband ever attended school	91.5 (989)	91.9 (431)	91.2 (558)	0.674
Average monthly household income [mean (SD)]	5,445.2 (7,438.9)	6,053.9 (7,688.4)	4,978.8 (7,213.5)	0.018
Caste or Tribe				
Scheduled caste	3.8 (41)	2.8 (13)	4.6 (28)	0.071
Scheduled tribe	68.0 (735)	65.5 (307)	69.9 (428)	
Other backward class	24.3 (263)	27.7 (130)	21.7 (133)	
None/Other	3.9 (42)	4.1 (19)	3.8 (23)	
Number of live births				
0	22.2 (240)	24.0 (112)	20.9 (128)	0.042
1	45.1 (488)	46.1 (216)	44.4 (272)	
2	24.0 (259)	20.0 (94)	27.0 (165)	
3+	8.7 (94)	10.0 (47)	7.7 (47)	
Number of living children				
0	23.1 (250)	24.7 (116)	21.9 (134)	0.081
1	47.1 (509)	47.3 (222)	46.9 (287)	
2	23.2 (251)	20.0 (94)	25.7 (157)	
3+	6.6 (71)	7.9 (37)	5.6 (34)	
Number of living sons				
0	55.5 (600)	58.0 (271)	54.0 (329)	0.098
1	38.3 (414)	35.0 (164)	40.9 (250)	
2+	6.2 (67)	50.8 (34)	5.4 (33)	
Number of living daughters				
0	52.6 (569)	53.7 (252)	51.8 (317)	0.800
1	34.5 (373)	33.5 (157)	35.3 (216)	
2+	12.9 (139)	12.8 (60)	12.9 (79)	
OUTCOMES:				
Modern Contraceptive Use^2^	28.4 (246)	29.2 (112)	27.7 (134)	0.614
Marital Contraceptive Communication	42.3 (457)	43.1 (202)	41.7 (255)	0.643
Self-reported pregnancy	19.8 (214)	18.3 (86)	20.9 (128)	0.292
Combined pregnancy^3^	22.1 (239)	21.5 (101)	22.6 (138)	0.691
Physical IPV	10.6 (114)	9.2 (43)	11.6 (71)	0.197
Sexual IPV	3.7 (40)	2.8 (13)	4.4 (27)	0.157
Men’s Attitudes toward Physical IPV	63.5 (685)	62.2 (290)	64.5 (395)	0.435
Men’s Attitudes toward Sexual IPV	37.7 (408)	38.2 (179)	37.4 (229)	0.802

^1^p-values based on chi-square analyses for categorical variables and on t-tests for continuous variables.

^2^Assessed for non-pregnant women, via self-report, at each time point

^3^Assessed via self-report or HCG test

A significant time by treatment effect on contraceptive use was seen in intent to treat analyses (p = 0.02), and marginally significant differences by treatment group were seen for contraceptive use at 9 and 18 month follow-ups. (See Table 3) Further examination of the time by treatment effect revealed that contraceptive use in the intervention group increased significantly from baseline at both 9 month (AOR = 2.13, 95% CI = 1.53, 2.95) and 18 month (AOR = 2.61, 95% CI = 1.88, 3.61) follow ups, while the control group increased at 18 month follow up (AOR = 1.51, 95% CI = 1.12, 2.04), but less than that seen in the intervention group. Dose analyses further revealed that contraceptive use doubled by 18 month follow-up among those participating in male only sessions (AOR = 1.96, 95% CI = 1.18, 3.27) and in male and couple sessions (AOR = 2.00, 95% CI = 1.26, 3.17), relative to those receiving no intervention sessions. (See Table 4) As seen in S1 Table, improvements in contraceptive use for intervention relative to control participants are largely attributable to condom use rather than other forms of contraceptives such as pill or female sterilization.

**Table 3:** Simple main effects for adjusted logistic GLMM assessing effects of CHARM intervention on modern contraceptive use, contraceptive communication, and pregnancy; per intent to treat (N = 1081 couples).

	Intervention	Control	Intervention vs. Control	Time x Group Interaction	Intracluster Correlation Coefficient^1^
	(n = 469)	(n = 612)			
	% (n)	% (n)	AOR*^2^ (95% CI)	p-value	P
Modern Contraceptive Use^3^				0.023	0.098
Baseline	29.2 (112)	27.7 (134)	0.92 (0.58–1.46)		
9 month	47.1 (160)	35.1 (163)	1.57 (0.995–2.49)		
18 month	51.7 (188)	39.8 (186)	1.58 (0.999–2.50)		
Marital Contraceptive Communication				0.002	0.062
Baseline	43.1 (202)	41.7 (255)	0.89 (0.62–1.29)		
9 month	49.9 (204)	34.9 (186)	1.77 (1.20–2.59)		
18 month	44.8 (188)	36.5 (194)	1.31 (0.89–1.94)		
Self-reported Pregnancy				0.150	-
Baseline	18.3 (86)	20.9 (128)	0.83 (0.60–1.14)		
9 month	16.4 (67)	12.6 (67)	1.36 (0.92–2.00)		
18 month	13.3 (56)	12.1 (64)	0.95 (0.62–1.47)		
Combined Pregnancy^4^				0.696	-
Baseline	21.5 (101)	22.6 (138)	0.93 (0.68–1.26)		
18 month	15.7 (66)	13.9 (74)	1.03 (0.69–1.53)		

* AOR: Adjusted Odds Ratio; 95% CI: 95% Confidence Interval

^1^ Cluster level correlation coefficient adjusted for all variables in the models; cluster not included as a random effect in pregnancy models due to estimated correlation equal to zero

^2^ Adjusted for wife’s age, wife’s education, caste/tribe, number of living sons, number of living daughters, and pregnancy intent

^3^ Assessed for non-pregnant women, via self-report, at each time point

^4^ Assessed via self-report or HCG test

**Table 4:** Simple main effects for adjusted logistic GLMM assessing effects of CHARM intervention on modern contraceptive use, contraceptive communication, and pregnancy; by actual session participation (N = 1081 couples).

	Male and Couple	Male Only	No sessions	Male and Couple vs. No sessions	Male only vs. No sessions	Time x Group Interaction	Intracluster Correlation Coefficient^1^
	(n = 246)	(n = 182)	(n = 653)				
	% (n)	% (n)	% (n)	AOR*^2^ (95% CI)	AOR*^1^ (95% CI)	p-value	P
Modern Contraceptive Use^3^						0.060	0.098
Baseline	31.3 (63)	26.9 (40)	27.7 (143)	1.10 (0.69–1.75)	0.99 (0.59–1.67)		
9 month	50.8 (95)	43.0 (55)	35.3 (173)	1.94 (1.23–3.07)	1.62 (0.97–2.71)		
18 month	55.0 (109)	51.1 (71)	39.4 (194)	2.00 (1.26–3.17)	1.96 (1.18–3.27)		
Marital Contraceptive Communication						0.002	0.063
Baseline	45.9 (113)	39.0 (71)	41.8 (273)	1.04 (0.71–1.52)	0.76 (0.50–1.16)		
9 month	56.0 (126)	42.9 (66)	35.2 (198)	2.35 (1.58–3.49)	1.31 (0.84–2.05)		
18 month	46.0 (104)	45.4 (74)	36.3 (204)	1.49 (0.93–2.08)	1.42 (0.90–2.23)		
Self-reported Pregnancy						0.499	-
Baseline	18.3 (45)	18.1 (33)	20.8 (136)	0.86 (0.58–1.28)	0.79 (0.51–1.24)		
9 month	16.1 (36)	16.9 (26)	12.8 (72)	1.35 (0.85–2.13)	1.28 (0.76–2.16)		
18 month	12.0 (27)	14.7 (24)	12.3 (69)	0.96 (0.57–1.61)	0.84 (0.46–1.52)		
Combined Pregnancy^4^						0.706	-
Baseline	22.0 (54)	19.8 (36)	22.8 (149)	0.96 (0.66–1.39)	0.80 (0.52–1.23)		
18 month	13.3 (30)	18.4 (30)	14.2 (80)	0.91 (0.56–1.47)	1.03 (0.61–1.75)		

* AOR: Adjusted Odds Ratio; 95% CI: 95% Confidence Interval

^1^ Cluster level correlation coefficient adjusted for all variables in the models; cluster not included as a random effect in pregnancy models due to estimated correlation equal to zero

^2^ Adjusted for wife’s age, wife’s education, caste/tribe, number of living sons, number of living daughters, and pregnancy intent

^3^ Assessed for non-pregnant women, via self-report, at each time point

^4^ Assessed via self-report or HCG test

A significant time by treatment effect on marital contraceptive communication was also demonstrated (p = 0.002). (See Table 3) Significant differences between treatment groups are seen for marital contraceptive communication at 9 month follow-up (AOR = 1.77, 95% CI = 1.20, 2.59) but not 18 month follow-up. Dose analyses further document that these observed effects are specific to participants receiving couple sessions (AOR = 2.35, 95% CI = 1.58, 3.49) relative to no sessions. Among participants who had not discussed contraception with their spouse at baseline, intervention participants were more likely to initiate a first discussion at 9 month follow-up than controls. (See S2 Table) Among participants who had discussed contraception with their spouse, intervention participants were more likely than control participants to continue those discussions at both 9 and 18 month follow-up.

No significant time by treatment effects were seen for pregnancy based on self-report or self-report combined with urine test results (p>0.15) within intent to treat analyses or by dose (see Tables 2 and 3) Exploratory analyses were conducted to assess if there were differences by treatment group and by dose for the outcomes of pregnancy or unintended pregnancy at either follow-up time point via chi-square analyses. No effect was observed for pregnancy, but the intervention group was significantly more likely (p = 0.02) to report having an unintended pregnancy over the follow-up period (15.2% vs. 10.1%). (See S3 Table) No significant difference in unintended pregnancy between groups was observed in dose analyses.

No significant time by treatment effect was seen for physical IPV. (See Table 5) However, significant time by treatment effects were observed for attitudes toward acceptability of physical IPV outcome (p = 0.01), with intervention men significantly less likely than controls to report attitudes of acceptability toward physical IPV at 18 month follow-up (AOR = 0.64, 95% CI = 0.44, 0.94). Dose analyses further demonstrate that men receiving male and couple sessions relative to no sessions were significantly less likely to be accepting of physical IPV at 18 month follow-up (AOR = 0.55, 95% CI = 0.37, 0.82). (See Table 6)

**Table 5:** Simple main effects for logistic GLMM assessing effect of CHARM intervention on physical IPV, sexual IPV, and men’s attitudes of acceptability towards physical IPV and sexual IPV; per intent to treat (N = 1081 couples).

	Intervention	Control	Intervention vs. Control	Time x Group Interaction	Intracluster Correlation Coefficient^1^
	% (n)	% (n)	AOR^2^ (95% CI)	p-value	P
Physical IPV^2^				0.730	0.046
Baseline	9.2 (43)	11.6 (71)	0.85 (0.53–1.34)		
9 month	14.2 (58)	19.5 (104)	0.75 (0.50–1.15)		
18 month	13.3 (56)	19.6 (104)	0.68 (0.44–1.06)		
Sexual IPV^2^				0.270	0.036
Baseline	2.8 (13)	4.4 (27)	0.71 (0.36–1.41)		
9 month	4.9 (20)	6.2 (33)	0.91 (0.50–1.65)		
18 month	5.0 (21)	10.8 (57)	0.48 (0.27–0.86)		
Men’s Attitudes Toward Physical IPV^3^				0.098	0.060
Baseline	62.2 (290)	64.5 (395)	0.96 (0.67–1.39)		
9 month	40.0 (161)	48.5 (250)	0.75 (0.51–1.09)		
18 month	36.8 (150)	49.1 (248)	0.64 (0.44–0.94)		
Men’s Attitudes Toward Physical IPV^3^				0.001	0.029
Baseline	37.9 (176)	37.2 (227)	1.09 (0.80–1.49)		
9 month	13.4 (54)	20.6 (106)	0.64 (0.43–0.95)		
18 month	8.3 (34)	16.0 (81)	0.51 (0.32–0.80)		

^1^ Cluster level correlation coefficient, adjusted for all variables in the models

^2^ IPV behavior models utilized women’s data and adjusted for wife’s age, wife’s education, caste/tribe, number of living sons, and number of living daughters

^3^IPV attitude models utilized men’s data and adjusted for husband’s age, husband’s education, and caste/tribe.

**Table 6:** Simple main effects for logistic GLMM assessing effect of CHARM intervention on physical IPV, sexual IPV, and men’s attitudes of acceptability towards physical IPV and sexual IPV; by actual session participation (N = 1081 couples).

	Male and Couple	Male Only	No sessions	Male and Couple vs. No sessions	Male only vs. No sessions	Time x Group Interaction	Intracluster Correlation Coefficient^1^
	(n = 246)	(n = 182)	(n = 653)				
	% (n)	% (n)	% (n)	AOR^1^ (95% CI)	AOR^1^ (95% CI)	p-value	P
Physical IPV^2^						0.962	0.045
Baseline	8.9 (22)	8.2 (15)	11.8 (77)	0.82 (0.48–1.40)	0.77 (0.42–1.42)		
9 month	13.8 (31)	14.9 (131)	19.2 (108)	0.77 (0.48–1.24)	0.85 (0.49–1.45)		
18 month	13.7 (31)	12.3 (20)	19.4 (109)	0.73 (0.44–1.20)	0.65 (0.36–1.17)		
Sexual IPV^2^						0.276	0.039
Baseline	2.9 (7)	2.2 (4)	4.4 (29)	0.70 (0.31–1.62)	0.58 (0.20–1.64)		
9 month	4.9 (11)	5.2 (8)	6.0 (34)	0.91 (0.45–1.84)	1.04 (0.47–2.31)		
18 month	6.6 (15)	2.5 (4)	10.5 (59)	0.70 (0.37–1.31)	0.22 (0.07–0.70)		
Men’s Attitudes toward Physical IPV^3^						0.093	0.059
Baseline	63.7 (156)	59.7 (108)	64.6 (421)	1.03 (0.70–1.50)	0.87 (0.58–1.31)		
9 month	37.8 (85)	42.7 (64)	48.3 (262)	0.68 (0.46–1.01)	0.86 (0.56–1.32)		
18 month	33.8 (74)	37.7 (60)	49.4 (264)	0.55 (0.37–0.82)	0.67 (0.43–1.03)		
Men’s Attitudes toward Sexual IPV^3^						0.026	0.029
Baseline	38.8 (95)	36.1 (65)	37.3 (243)	1.17 (0.83–1.65)	1.02 (0.69–1.49)		
9 month	13.3 (30)	12.8 (19)	20.5 (111)	0.66 (0.41–1.04)	0.60 (0.35–1.04)		
18 month	9.1 (20)	7.6 (12)	15.5 (83)	0.61 (0.35–1.04)	0.47 (0.25–0.91)		

^1^Cluster level correlation coefficient, adjusted for all variables in the models.

^2^IPV behavior models utilized women’s data and adjusted for wife’s age, wife’s education, caste/tribe, number of living sons, and number of living daughters

^3^IPV attitude models utilized men’s data and adjusted for husband’s age, husband’s education and caste/tribe

No significant time by treatment effects were seen for sexual IPV. (See Table 5) However, women in the intervention group were significantly less likely than controls to report sexual IPV at 18 month follow-up (AOR = 0.48, 95% CI = 0.27, 0.86). Significant time by treatment interaction effects were seen for attitudes accepting of sexual IPV (p<0.001), with intervention men significantly less likely than controls to report acceptance of sexual IPV at 9 month (AOR = 0.64, 95% CI = 0.43, 0.95) and 18 month follow-ups (AOR = 0.51, 95% CI = 0.32, 0.80). Small cell sizes resulted in unstable estimates for sexual IPV and sexual IPV attitudes in dose analyses. (See Table 6)

Men who received CHARM and participated in the response to program survey (n = 347) largely (>93%) reported comprehensive FP content coverage and positive response to program, but only half of participants reported receipt of contraceptives, almost exclusively in the form of condoms. (See Table 7) Most participants (81.6%) found the men’s sessions very useful, but only 58.5% found the couple session useful. The majority “very much” enjoyed the program (85.3%) and “very much” felt it should be continued (78.1%).

**Table 7:** Male Participant Experiences of CHARM Intervention Delivery and Satisfaction with the Program (n = 347 men who participated in at least one CHARM session).

Questions	n (%)
Topics Covered by village health providers (Not mutually exclusive)	
Condoms	347 (100.0)
IUD	328 (94.5)
Oral contraceptive pills	343 (98.8)
Family size (number of children)	340 (98.0)
Spacing between the children	340 (98.0)
Family Planning marital communication	339 (97.7)
Joint marital decision-making on family planning	338 (97.4)
Contraceptive use	346 (99.7)
Delaying pregnancy	346 (99.7)
Availability and accessibility of spacing contraceptive methods	325 (93.7)
Received Contraceptives from the village health provider	
Yes, More than Once	25.6 (89)
Yes, Once	24.2 (84)
No	50.1 (174)
Form of Contraception Received from village health providers (not mutually exclusive)	
Condom	48.1 (167)
Oral Contraceptive Pill	1.7 (6)
IUD	0.3 (1)
Perception of village health providers’ Family Planning Knowledge	
Very Knowledgeable	88.2 (306)
Somewhat Knowledgeable	11.5 (40)
Not at All Knowledgeable	0.3 (1)
Felt the Men’s Sessions were Important	
Very Much	81.6 (283)
Somewhat	18.2 (63)
Not at All	0.3 (1)
Felt the Couples’ Sessions were Important	
Very Much	58.5 (203)
Somewhat	18.7 (65)
Not at All	2.3 (8)
Enjoyed the CHARM Program	
Very Much	85.3 (296)
Somewhat	14.2 (49)
Not at All	0.6 (2)
Recommend that the CHARM Program be Continued	
Very Much	78.1 (271)
Somewhat	21.0 (73)
Not at All	0.9 (3)

Note: Responses are mutually exclusive unless otherwise noted in table.

## Discussion

Study findings document the effectiveness of CHARM in engaging husbands and improving marital contraceptive communication and use among young couples in rural Maharashtra, India. These findings build on prior research demonstrating that men can be effectively engaged to promote family planning in their marital relationships [27–31]. Dose analyses further reveal that couple sessions were required to improve contraceptive communication, highlighting the importance of couple sessions, despite the greater difficulty in achieving couple versus male only session participation (52% vs 91%). Nonetheless, even male-only sessions show promise in improving marital contraceptive use in this rural population in India.

Results of this study also demonstrate effects of CHARM on reduction in men’s perpetration of sexual IPV and attitudes toward acceptability of IPV, likely due to inclusion of GE counseling. Prior research from Africa indicates that GE counseling with men, alone or in the context of HIV prevention, reduces IPV perpetration [32–35]; the current study extends these findings to FP programs and South Asia. Notably, increases in physical and sexual IPV were reported for the control condition, and in physical IPV for the CHARM condition. It is unclear whether this is due to increases in violence or greater disclosure over time. Field reports suggest increased disclosure occurred, as rapport was built with research staff collecting data over the course of the study. Further research is needed to confirm this observation.

The CHARM intervention did not demonstrate impact on pregnancy. Relative lack of impact on incident pregnancy may be due to CHARM effects on contraception largely being in the form of condom use, which is not a highly effective form of contraception, given its 21% failure rate [36]. Exploratory analyses on unintended pregnancy reinforce these findings, with unintended pregnancy being more likely among intervention relative to control participants over time. Future replication of CHARM would benefit from prioritization of more effective contraceptive methods, such as intrauterine devices, and better engagement of wives to facilitate their use.

While study results are promising, they must be considered in light of certain limitations. Outcomes were largely reliant on self-report, and are vulnerable to recall and social desirability biases. Our primary outcome of contraceptive use does not include consideration of consistency in use. Low reporting on some outcomes, such as items related to acceptability of sexual violence, may affect findings, particularly for dose analyses. Additionally, fidelity to intervention also relied on self-report. Ideally, audiotapes or direct observations would have been used, but this was not preferred by participants due to the sensitivity of the topics discussed. There were also difference between groups on women’s education and income at baseline, despite randomization, but there were no differences in any of the outcomes assessed in this study. The study was conducted in a single Indian district in one state, limiting generalizability. The unintended pregnancy variable relies on intention as reported 9 months prior to the pregnancy, and intention to become pregnant may have changed in that period. While CHARM coverage of FP-related information was high, lack of assessment regarding GE-related information impedes our understanding of quality and consistency of delivery of those components. We must also note that the evaluation leadership was involved with the development of the intervention. However, the medical college overseeing training and supervision of the CHARM providers, and the providers themselves, were not part of the evaluation team. Finally, the study did not assess indicators of sustainability, such as cost-effectiveness or investment opportunities for sustainability; further work is needed to determine whether broad adoption and implementation of CHARM could be undertaken in rural communities across India.

## Conclusion

The CHARM intervention, which involves three GE+FP counseling sessions delivered by male health care providers to married men, alone (sessions 1&2) and with their wives (session 3) over three months, appears to be an effective approach to engage men in family planning, improve marital contraceptive communication and use, and reduce male perpetration of sexual IPV. This low intensity intervention, able to be delivered by existing rural medical providers, including non-allopathic private providers, offers a potentially sustainable approach to improve spacing contraceptive use among young couples in rural India and possibly elsewhere. The opportunity to involve the private informal sector for this effort in rural India cannot be understated, given that the majority of the primary care in rural India comes from this sector but family planning has not been within their purview [2, 37, 38]. The intervention may further benefit from more expanded choices for contraceptives and better reach to women for use of more effective contraceptives, given that the current model did not affect highly effective contraceptive use or incident pregnancy. Greater efforts to include women and provide more effective contraceptives (e.g., intrauterine device- IUD) in future may be facilitated by recent government commitments to support an expansion of more effective contraceptive options in India and ensure universal access to family planning services [39].

Prioritization of innovative approaches such as CHARM to improve family planning in India is timely, as recently released data from multiple Indian states reveal no improvement in contraceptive uptake across the past decade, with some states even demonstrating a decline in modern contraceptive use in this timeframe [40]. These same data further indicate that health worker outreach to women for family planning promotion has improved in many states, but with no corresponding improvements in contraceptive uptake [40]. Engaging men as partners to support family planning may be a key to turning the tide in the country. The evidence-based CHARM intervention, which capitalizes on the country’s existing rural health infrastructure inclusive of the numerous and accessible private providers, may offer an important means to accelerate improvements in family planning uptake and help India achieve its FP2020 goal of creating 40 million new users of contraception by 2020 [41].

## Supporting Information

S1 Table Frequency of specific contraception use in the past 3 months^1^ for whole sample and by group (N = 1,081). (DOCX)

S2 Table Percentage of women reporting spousal discussion on contraception in the previous 3 months for whole sample and by group^1^ (N = 1081). (DOCX)

S3 Table Percentage of women reporting at least one pregnancy and unintended pregnancy at either follow-up time point, for whole sample, by group^1^, and by actual session attendance^1^ (N = 1081). (DOCX)
