# Supplementary material for: Cluster Randomized Controlled Trial Evaluation of a Gender Equity and Family Planning Intervention for Married Men and Couples in Rural India
Source: PLoS One. 2016 May 11;11(5):e0153190. doi: 10.1371/journal.pone.0153190 (PMC4864357; doi:10.1371/journal.pone.0153190)
Supplement: S1 Table — (DOCX) [file pone.0153190.s001.docx]

**S1 Table. Frequency of specific contraception use in the past 3 months^1^ for whole sample and by group (N=1,081)**

|  | **T1: Baseline** | | | **T2: 9 Month Follow up** | | | **T3: 18 Month Follow up** | | |
| --- | --- | --- | --- | --- | --- | --- | --- | --- | --- |
|  | Total Sample | Intervention | Control | Total Sample | Intervention | Control | Total Sample | Intervention | Control |
|  | (n=867) | (n=383) | (n=484) | (n=805) | (n=340) | (n=465) | (n=831) | (n=364) | (n=467) |
| Method^2^ | % (n) | % (n) | % (n) | % (n) | % (n) | % (n) | % (n) | % (n) | % (n) |
| Male condom | 14.4 (125) | 16.4 (63) | 12.8 (62) | 20.5 (165) | 29.1 (99) | 14.2 (66) | 19.6 (163) | 25.0 (91) | 15.4 (72) |
| IUD | 2.3 (20) | 2.9 (11) | 1.9 (9) | 2.1 (17) | 2.4 (8) | 1.9 (9) | 2.3 (19) | 2.7 (10) | 1.9 (9) |
| Pill | 12.3 (107) | 10.4 (40) | 13.8 (67) | 10.3 (83) | 8.8 (30) | 11.4 (53) | 9.4 (78) | 8.5 (31) | 10.1 (47) |
| Female Sterilization | n/a | n/a | n/a | 7.3 (59) | 7.4 (25) | 7.3 (34) | 14.1 (117) | 15.0 (55) | 13.5 (62) |
| Injectables | 0.1 (1) | 0.3 (1) | 0 (0) | 0.7 (6) | 0.6 (2) | 0.9 (4) | 0.5 (4) | 0.5 (2) | 0.4 (2) |
| No Modern Contraception | 71.6 (621) | 70.8 (271) | 72.3 (350) | 59.9 (482) | 52.9 (180) | 65.0 (302) | 55.0 (457) | 48.4 (176) | 60.2 (281) |

1 Assessed for women self-reporting as non-pregnant at each time point

2 Women could select for more than one method
