# Supplementary material for: Cluster Randomized Controlled Trial Evaluation of a Gender Equity and Family Planning Intervention for Married Men and Couples in Rural India
Source: PLoS One. 2016 May 11;11(5):e0153190. doi: 10.1371/journal.pone.0153190 (PMC4864357; doi:10.1371/journal.pone.0153190)
Supplement: S2 Table — (DOCX) [file pone.0153190.s002.docx]

**S2 Table. Percentage of women reporting contraceptive communication in the previous 3 months for whole sample and by group^1^ (N=1081)**

|  | Frequencies | | | New Communication | | | Continuing Communication | |
| --- | --- | --- | --- | --- | --- | --- | --- | --- |
|  | Baseline | 9 month | 18 month | 9 month but not at baseline | 18 month but not at baseline or 9 month | 9 or 18 month but not at baseline | Baseline and 9 or 18 month | Baseline, 9, and 18 months (all 3 time points) |
|  | % (n) | % (n) | % (n) | % (n) | % (n) | % (n) | % (n) | % (n) |
| Total Sample | 41.7 (255) | 34.9 (186) | 36.5 (194) | 19.3 (182) | 10.7 (93) | 28.3 (289) | 33.4 (301) | 12.2 (106) |
| Intervention | 43.1 (202) | **49.9 (204)** | **44.8 (188)** | **23.7 (97)** | 10.7 (41) | **32.7 (146)** | **37.9 (150)** | **14.6 (56)** |
| Control | 41.7 (255) | **34.9 (186)** | **36.5 (194)** | **16.0 (85)** | 10.7 (52) | **24.8 (143)** | **29.9 (151)** | **10.3 (50)** |

Note: Bolded text indicates significant difference between CHARM and control group at p ≤ 0.05, based on chi-square analyses
