# Supplementary material for: Cluster Randomized Controlled Trial Evaluation of a Gender Equity and Family Planning Intervention for Married Men and Couples in Rural India
Source: PLoS One. 2016 May 11;11(5):e0153190. doi: 10.1371/journal.pone.0153190 (PMC4864357; doi:10.1371/journal.pone.0153190)
Supplement: S3 Table — (DOCX) [file pone.0153190.s003.docx]

**S3 Table. Percentage of women reporting at least one pregnancy and unintended pregnancy at either follow-up time point, for whole sample, by group1 and by actual session attendance1 (N=1,081)**

|  | **Any Pregnancy %(n)** | **Any Unintended Pregnancy** |
| --- | --- | --- |
|  |  | **% (n)** |
| Total Sample | 30.5(272) | 12.3(107) |
|  |  |  |
| Intervention | 33.4(132) | **15.2(58)** |
| Control | 28.1(140) | **10.1(49)** |
|  |  |  |
| No Sessions | 28.6(150) | 10.4(53) |
| Only Male Sessions | 36.8(56) | 13.8(20) |
| Male and Couple Sessions | 30.4(66) | 16.1(34) |

Note: Bolded text indicates significant difference between groups at p<0.05, based on chi-square analyses.
